# Supplementary material for: Whole-Genome Sequencing of Linezolid-Resistant and Linezolid-Intermediate-Susceptibility Enterococcus faecalis Clinical Isolates in a Mexican Tertiary Care University Hospital
Source: Microorganisms. 2025 Mar 19;13(3):684. doi: 10.3390/microorganisms13030684 (PMC11944505; doi:10.3390/microorganisms13030684)

SUPPLEMENTARY MATERIALS

Table S1. Sequencing quality metrics for linezolid non-susceptible E. faecalis isolates (n = 24)
Table S2. Quality Metrics of *Enterococcus faecalis* Genome Assemblies
Table S3. Clinical and microbiological characteristics of patients with *Enterococcus faecalis* non-susceptible to linezolid
Table S4. Identification of antibiotic resistance proteins using BLASTP analysis in clinical isolates.
Table S5. Virulence factor profiles of *E. faecalis* isolates by clinical source and MLST
Figure S1. Antibiotic resistance profile of LNSEf isolates

Table S1. Sequencing quality metrics for linezolid non-susceptible *E. faecalis* isolates (n = 24)

Label	ID Sample	Clean reads	Clean bases	Q30%	GC%
Ef-1	5923	1,446,970	199,839,962	94.61	38.81
Ef-2	6224	2,193,402	312,248,519	97.36	37.56
Ef-3	6463	2,488,978	358,317,815	94.30	38.59
Ef-4	6871	1,306,208	186,958,794	96.40	37.66
Ef-5	7317	2,459,832	356,993,810	96.59	37.59
Ef-6	7525	1,958,530	284,338,695	96.64	37.45
Ef-7	7659	3,848,142	561,225,675	96.61	37.18
Ef-8	7779	2,493,640	363,253,630	96.50	37.28
Ef-9	9077	2,713,824	1396,317,170	96.63	37.45
Ef-10	9271	3,535,782	510,216,531	96.76	37.34
Ef-11	9425	2,287,526	326,872,462	95.46	37.98
Ef-12	9435	4,875,968	713,238,077	95.11	38.3
Ef-13	9667	2,116,478	302,190,433	96.53	37.55
Ef-14	9732	10,093,018	1,456,037,000	95.72	37.55
Ef-15	9946	3,990,328	582,609,050	96.76	37.40
Ef-16	10759	3,795,982	555,770,291	96.93	37.50
Ef-17	10815	1,030,056	140,850,197	97.04	38.33
Ef-18	10890	1,050,666	145,203,434	96.93	37.19
Ef-19	11984	3,243,758	468,972,339	96.8	37.33
Ef-20	12065	1,707,810	242,016,073	97.08	38.03
Ef-21	12080	2,351,322	335,736,856	96.78	37.45
Ef-22	12139	2,540,676	368,925,820	96.73	37.64
Ef-23	12173	2,433,878	356,595,077	96.61	37.63
Ef-24	12417	3,977,488	578,966,182	96.79	37.17

Table S2. Quality metrics of *Enterococcus faecalis* genome assemblies

Label	ID Sample	Accession	Contigs	Total Length	N50 (bp)	L50	Longest Contig (bp)	GC Content (%)
Ef-1	5923	JBLVLD000000000	46	282,6108	122, 882	8	26,7026	37.57
Ef-2	6224	JBLIYG000000000	56	297,9826	140,803	6	48,8300	37.30
Ef-3	6463	JBLIYF000000000	26	288,8683	283,835	3	895,528	37.35
Ef-4	6871	JBLIYE000000000	24	279,2195	306,797	3	797,479	37.55
Ef-5	7317	JBLVLC000000000	31	2,816,181	272,070	4	576,941	37.56
Ef-6	7525	JBLVLB000000000	89	2,999,824	185,572	5	563,126	37.25
Ef-7	7659	JBLIYD000000000	29	2,900,283	282,503	4	464, 220	37.34
Ef-8	7779	JBLVLA000000000	29	2,894,943	294,654	4	564,277	37.51
Ef-9	9077	JBLIYC000000000	32	2,879,819	313,695	4	39,2151	37.51
Ef-10	9271	JBLVKZ000000000	47	2,879,494	132,142	6	603,810	37.47
Ef-11	9425	JBLIYB000000000	22	2,821,924	277,805	3	971,957	37.52
Ef-12	9435	JBLIYA000000000	27	2,789,367	604,658	2	860,184	37.54
Ef-13	9667	JBLVKY000000000	40	2,963,641	183,737	6	544,846	37.37
Ef-14	9732	JBLIXZ000000000	35	2,921,377	347,540	4	498,005	37.24
Ef-15	9946	JBLIXY000000000	37	3,096,506	322,890	4	529,489	37.15
Ef-16	10759	JBLIXX000000000	26	2,884,669	282,497	4	463,994	37.35
Ef-17	10815	JBLIXW000000000	95	2,787,254	46,611	18	176,640	37.56
Ef-18	10890	JBLIXV000000000	56	2,987,184	152,992	7	393,803	37.22
Ef-19	11984	JBLIXU000000000	60	3,127,719	158,082	7	429,113	37.19
Ef-20	12065	JBLIXT000000000	89	2812,674	62,267	15	162,927	37.53
Ef-21	12080	JBLIXS000000000	39	2,885,374	151,109	7	463,871	37.34
Ef-22	12139	JBLIXR000000000	37	2,811,482	194,843	5	546,435	37.51
Ef-23	12173	JBLIXQ000000000	39	2,814,746	268,300	4	564,494	37.55
Ef-24	12417	JBLVKX000000000	42	3, 121,764	197,324	6	412,395	37.21

Table S3. Clinical and microbiological characteristics of patients with *Enterococcus faecalis* non-susceptible to linezolid

Label	Date	Source	Sample Type	Occupation	Sex	Age	Previous antibiotic use <90 days	Previous hospitalization <90 days	Charlson comorbidity index	Previous surgical history <90 days	Central Catheter	Urinary Catheter	Initial Empirical Treatment	Hospital Stay (Days)	Outcome
Ef-1	Jul-23	Traumatology	Surgical wound secretion	Delivery worker	Male	28	Present	Absent	0	Present	Absent	Present	CRO+CLI	27	Discharge
Ef-2	Aug-23	Traumatology	Surgical wound secretion	Unemployed	Male	63	Absent	Present	3	Present	Absent	Present	CZ	39	Discharge
Ef-3	Aug-23	Cardiology	Urine	Housewife	Female	53	Absent	Absent	1	Absent	Present	Present	TZP	2	Death
Ef-4	Sep-23	General Surgery	Abscess	Commerce	Male	43	Absent	Absent	0	Present	Absent	Absent	AN+MTZ	12	Discharge
Ef-5	Oct-23	Infectious Diseases	Tissue	Housewife	Female	55	Present	Present	3	Absent	Absent	Present	CRO+CLI	14	Discharge
Ef-6	Oct-23	Pediatrics	Surgical wound	Student	Male	14	Present	Absent	0	Absent	Present	Present	VAN	15	Discharge
Ef-7	Oct-23	Internal Medicine	Peritoneal fluid	Unemployed	Male	57	Present	Present	2	Present	Present	Absent	CRO+MTZ	4	Discharge
Ef-8	Oct-23	General Surgery	Surgical wound	Construction	Male	49	Present	Present	0	Present	Absent	Present	TZP	8	Discharge
Ef-9	Dec-23	Gastroenterology	Urine	Housewife	Female	64	Absent	Present	4	Present	Absent	Present	CRO	9	Discharge
Ef-10	Dec-23	Nephrology	Urine	Unemployed	Male	73	Absent	Present	5	Present	Absent	Present	Not considered for treatment	17	Death
Ef-11	Jan-24	Plastic Surgery	Skin tissue	Unemployed	Female	34	Absent	Absent	0	Present	Present	Present	Not considered for treatment	3	Discharge
Ef-12	Jan-24	Urology	Urine	Courier	Male	55	Absent	Present	1	Present	Absent	Present	CRO	3	Discharge
Ef-13	Jan-24	Thoracic and Cardiovascular Surgery	Foot tissue	Unemployed	Male	55	Absent	Present	1	Present	Absent	Absent	CRO+CLI	14	Discharge
Ef-14	Jan-24	Thoracic and Cardiovascular Surgery	Foot tissue	Housewife	Female	44	Present	Present	1	Present	Absent	Absent	AMX	20	Discharge
Ef-15	Feb-24	Hematology	Central blood culture	Engineer	Female	65	Present	Present	6	Absent	Present	Present	MEM+LNZ	14	Death
Ef-16	Mar-24	HIV Unit	Peripheral blood culture	Construction	Male	39	Absent	Absent	8	Absent	Present	Present	LNZ	13	Discharge
Ef-17	Mar-24	General Surgery	Peritoneal fluid	Student	Male	27	Present	Absent	0	Absent	Present	Present	TZP+MTZ	13	Discharge
Ef-18	Mar-24	Transplant Unit	Urine	Unemployed	Male	32	Present	Present	2	Present	Absent	Absent	CTX	23	Discharge
Ef-19	May-24	Infectious Diseases	Urine	Unemployed	Male	1	Absent	Present	0	Present	Absent	Absent	Not considered for treatment	29	Discharge
Ef-20	Jun-24	Traumatology	Femur	Student	Male	24	Present	Absent	0	Absent	Absent	Present	Not considered for treatment	21	Discharge
Ef-21	Jun-24	Cardiology	Urine	Housewife	Female	57	Absent	Absent	3	Absent	Present	Present	Not considered	15	Death

													for treatment		
Ef-22	Jun-24	Plastic Surgery	Central blood culture	Student	Male	23	Present	Absent	0	Present	Present	Present	CAZ+VAN	12	Discharge
Ef-23	Jun-24	Thoracic and Cardiovascular Surgery	Granuloma	Unemployed	Male	70	Absent	Absent	4	Present	Present	Absent	Not considered for treatment	23	Discharge
Ef-24	Jun-24	Neurosurgery	CSF	Unemployed	Male	47	Absent	Present	0	Present	Absent	Present	CRO	20	Discharge

Table S4. Identification of antibiotic resistance proteins using BLASTP analysis in clinical isolates.

Label	ID Sample	Query	Description	Scientific Name	Taxid	Max Score	Total Score	Query Cover	E-Value	Percentage Identity	Accession
Ef-1	5923	optrA_2_KT862781_1	ABC-F type ribosomal protection protein OptrA	Enterococcus faecalis	1351	64.3	64.3	100%	6.00E-10	95.00%	NSN46193.1
Ef-2	6224	optrA_7_KT862775_1	ABC-F type ribosomal protection protein OptrA	Enterococcus faecalis	1351	63.8	63.8	100%	8.00E-10	95.00%	WP_342834678.1
Ef-3	6463	optrA_15_KX620942_1	ABC-F type ribosomal protection protein OptrA	Enterococcus faecalis	1351	63.8	63.8	100%	8.00E-10	95.00%	WP_342834678.1
Ef-4	6871	optrA_5_KT862783_1	ABC-F type ribosomal protection protein OptrA	Enterococcus faecalis	1351	63.8	63.8	100%	8.00E-10	95.00%	WP_342834678.1
Ef-5	7317	optrA_5_KT862783_1	ABC-F type ribosomal protection protein OptrA	Enterococcus faecalis	1351	63.8	63.8	100%	8.00E-10	95.00%	WP_342834678.1
Ef-6	7525	optrA_5_KT862783_1	ABC-F type ribosomal protection protein OptrA	Enterococcus faecalis	1351	63.8	63.8	100%	8.00E-10	95.00%	WP_342834678.1
Ef-7	7659	optrA_5_KT862783_1	ABC-F type ribosomal protection protein OptrA	Enterococcus faecalis	1351	63.8	63.8	100%	8.00E-10	95.00%	WP_342834678.1
Ef-8	7779	optrA_2_KT862781_1	ABC-F type ribosomal protection protein OptrA	Enterococcus faecalis	1351	64.3	64.3	100%	6.00E-10	95.00%	NSN46193.1
Ef-9	9077	optrA_5_KT862783_1	ABC-F type ribosomal protection protein OptrA	Enterococcus faecalis	1351	63.8	63.8	100%	8.00E-10	95.00%	WP_342834678.1
Ef-10	9271	optrA_5_KT862783_1	ABC-F type ribosomal protection protein OptrA	Enterococcus faecalis	1351	63.8	63.8	100%	8.00E-10	95.00%	WP_342834678.1
Ef-11	9425	optrA_5_KT862783_1	ABC-F type ribosomal protection protein OptrA	Enterococcus faecalis	1351	63.8	63.8	100%	8.00E-10	95.00%	WP_342834678.1
Ef-12	9435	optrA_15_KX620942_1	ABC-F type ribosomal protection protein OptrA	Enterococcus faecalis	1351	63.8	63.8	100%	8.00E-10	95.00%	WP_342834678.1
Ef-13	9667	optrA_5_KT862783_1	ABC-F type ribosomal protection protein OptrA	Enterococcus faecalis	1351	63.8	63.8	100%	8.00E-10	95.00%	WP_342834678.1
Ef-14	9732	optrA_7_KT862775_1	ABC-F type ribosomal protection protein OptrA	Enterococcus faecalis	1351	63.8	63.8	100%	8.00E-10	95.00%	WP_342834678.1
Ef-15	9946	optrA_15_KX620942_1	ABC-F type ribosomal protection protein OptrA	Enterococcus faecalis	1351	63.8	63.8	100%	8.00E-10	95.00%	WP_342834678.1
Ef-16	10759	optrA_5_KT862783_1	ABC-F type ribosomal protection protein OptrA	Enterococcus faecalis	1351	63.8	63.8	100%	8.00E-10	95.00%	WP_342834678.1
Ef-17	10815	optrA_15_KX620942_1	ABC-F type ribosomal protection protein OptrA	Enterococcus faecalis	1351	63.8	63.8	100%	8.00E-10	95.00%	WP_342834678.1
Ef-18	10890	optrA_15_KX620942_1	ABC-F type ribosomal protection protein OptrA	Enterococcus faecalis	1351	63.8	63.8	100%	8.00E-10	95.00%	WP_342834678.1
Ef-19	11984	optrA_15_KX620942_1	ABC-F type ribosomal protection protein OptrA	Enterococcus faecalis	1351	63.8	63.8	100%	8.00E-10	95.00%	WP_342834678.1
Ef-20	12065	optrA_8_KT862782_1	ABC-F type ribosomal protection protein OptrA	Enterococcus faecalis	1351	63.8	63.8	100%	8.00E-10	95.00%	WP_342834678.1
Ef-21	12080	optrA_5_KT862783_1	ABC-F type ribosomal protection protein OptrA	Enterococcus faecalis	1351	63.8	63.8	100%	8.00E-10	95.00%	WP_342834678.1
Ef-22	12139	optrA_10_KT601170_1	ABC-F type ribosomal protection protein OptrA	Streptococcus suis	1307	65.1	65.1	100%	3.00E-10	95.00%	WP_256779199.1
Ef-23	12147	optrA_15_KX620942_1	ABC-F type ribosomal protection protein OptrA	Enterococcus faecalis	1351	63.8	63.8	100%	8.00E-10	95.00%	WP_342834678.1
Ef-24	12173	optrA_15_KX620942_1	ABC-F type ribosomal protection protein OptrA	Enterococcus faecalis	1351	63.8	63.8	100%	8.00E-10	95.00%	WP_342834678.1

Ef-20	**12065**	cfr_1_AM408573_1	23S rRNA (adenine(2503)-C(8))-methyltransferase Cfr	Staphylococcus aureus	1280	70.2	70.2	100%	4.00E-12	100.00%	MEZ2068495.1
Ef-22	**12139**	cfr_1_AM408573_1	23S rRNA (adenine(2503)-C(8))-methyltransferase Cfr	Staphylococcus aureus	1280	70.2	70.2	100%	4.00E-12	100.00%	MEZ2068495.1

Table S5. Virulence factor profiles of *E. faecalis* isolates by clinical source and MLST

Label	Date	Source	MLST	Virulence Factor
Ef-1	jul-23	Traumatology	ST101	*fss1, cpsA, cpsB, cpsC, cpsD, cpsE, cpsG, cpsH, cpsI, cpsJ, cpsK, fsrA, fsrB, fsrC, EF3023, ebpA, ebpB, ebpC, srtC, ace, sprE, gelE, EF0818, bopD, efaA.*
Ef-2	Aug-23	Traumatology	ST283	*srtC, ebpC, ebpB, ebpA, EF0485, fss3, efaA, bopD, fss1, cpsB, cpsA.*
Ef-3	Aug-23	Cardiology	ST585	*ebpA, ebpB, ebpC, srtC, cylI, cylA, cylB, cylM, cylS, cylL, cylR1, cylR2, cpsK, cpsJ, cpsI, cpsH, cpsG, cpsF, cpsE, cpsD, cpsC, cpsB, cpsA, fss2, sprE, gelE, fsrC, fsrB, fsrA, efaA, bopD, fss1, EF0485*
Ef-4	sep-23	General Surgery	ST506	*srtC, ebpC, ebpB, ebpA, EF3023, EF0818, efaA, fsrA, fsrB, fsrC, sprE, fss2, cpsA, cpsB, cpsC, cpsD, cpsE, cpsG, cpsH, cpsI, cpsJ, cpsK, bopD, fss1.*
Ef-5	oct-23	Infectious Diseases	ST476	*srtC, ebpC, ebpB, ebpA, efaA, bopD, fss2, cpsA, cpsB, cpsC, cpsD, cpsE, cpsF, cpsG, cpsH, cpsI, cpsJ, cpsK, EF3023, fss1.*
Ef-6	oct-23	Pediatrics	ST287	*fss1, srtC, ebpC, ebpB, ebpA, fsrA, fsrB, fsrC, gelE, sprE, EF0485, cylI, bopD, EF0818, fss2, cpsK, cpsJ, cpsI, cpsH, cpsG, cpsF, cpsE, cpsD, cpsC, cpsB, cpsA, efaA*
Ef-7	oct-23	Internal Medicine	ST585	*ebpA, ebpB, ebpC, srtC, cylI, cylA, cylB, cylM, cylS, cylL, cylR1, cylR2, cpsK, cpsJ, cpsI, cpsH, cpsG, cpsF, cpsE, cpsD, cpsC, cpsB, cpsA, fss2, sprE, gelE, fsrC, fsrB, fsrA, efaA, bopD, fss1, EF0485.*
Ef-8	oct-23	General Surgery	ST202	*srtC, ebpC, ebpB, ebpA, sprE, gelE, fsrC, fsrB, fsrA, efaA, cpsA, cpsB, ecbA, EF0818, bopD, fss1.*
Ef-9	Dec-23	Gastroenterology	ND	*prgB/asc10, sprE, gelE, fsrC, fsrB, fsrA, efaA, EF3023, cpsK, cpsJ, cpsI, cpsH, cpsG, cpsF, cpsE, cpsD, cpsC, cpsB, cpsA, fss2, bopD, fss1, ebpA, ebpB, ebpC, srtC.*
Ef-10	Dec-23	Nephrology	ST480	*srtC, ebpC, ebpB, ebpA, prgB/asc10, EF3023, bopD, cpsB, cpsA, fss1, efaA.*
Ef-11	Jan-24	Plastic Surgery	ST506	*EF0818, EF3023, efaA, fsrA, fsrB, fsrC, gelE, sprE, srtC, ebpC, ebpB, ebpA, fss2, cpsA, cpsB, cpsC, cpsD, cpsE, cpsG, cpsH, cpsI, cpsJ, cpsK, bopD, fss1.*
Ef-12	Jan-24	Urology	ST376	*efaA, fsrA, fsrB, fsrC, gelE, sprE, EF0818, bopD, fss2, cpsA, cpsB, cpsC, cpsD, cpsE, cpsG, cpsH, cpsI, cpsJ, cpsK, EF3023, fss1, ebpA, ebpB, ebpC, srtC, ace.*
Ef-13	Jan-24	Thoracic and Cardiovascular Surgery	ST179	*fss2, asa1, cylI, cpsA, cpsB, cpsC, cpsD, cpsE, cpsF, cpsG, cpsH, cpsI, cpsJ, cpsK, srtC, ebpC, ebpB, ebpA, cylA, cylB, cylM, cylS, cylL, cylR1, cylR2, efaA, bopD, EF0485, EF3023, fss1*
Ef-14	Jan-24	Thoracic and Cardiovascular Surgery	ST16	*cylI, cylA, cylB, cylM, cylS, cylL, cylR1, cylR2, bopD, efaA, EF3023, EF0485, cpsA, cpsB, cpsC, cpsD, cpsE, cpsF, cpsG, cpsH, cpsI, cpsJ, cpsK, fss1, srtC, ebpC, ebpB, ebpA.*
Ef-15	feb-24	Hematology	ST69	*fss1, cylI, cylA, cylB, cylM, cylS, cylL, cylR1, cylR2, sprE, gelE, fsrC, fsrB, fsrA, efaA, cpsA, cpsB, EF3023, EF0818, bopD, prgB/asc10, srtC, ebpC, ebpB, ebpA.*
Ef-16	mar-24	HIV Unit	ST585	*ebpA, ebpB, ebpC, srtC, cylI, cylA, cylB, cylM, cylS, cylL, cylR1, cylR2, cpsK, cpsJ, cpsI, cpsH, cpsG, cpsF, cpsE, cpsD, cpsC, cpsB, cpsA, fss2, sprE, gelE, fsrC, fsrB, fsrA, efaA, bopD, fss1, EF0485*
Ef-17	mar-24	General Surgery	ST32	*fss2, cpsA, cpsB, cpsC, cpsD, cpsE, cpsF, cpsG, cpsH, cpsI, cpsJ, cpsK, fsrA, fsrB, fsrC, gelE, sprE, srtC, ebpC, ebpB, ebpA, efaA, fss1, bopD.*
Ef-18	mar-24	Transplant Unit	ST415	*EF3023, efaA, ace, srtC, ebpC, ebpB, ebpA, EF0818, fsrA, fsrB, fsrC, gelE, sprE, cpsK, cpsJ, cpsI, cpsH, cpsG, cpsE, cpsD, cpsC, cpsB, cpsA, fss2, fss1, bopD*
Ef-19	may-24	Infectious Diseases	ST40	*fsrA, fsrB, fsrC, gelE, sprE, prgB/asc10, cylA, cylI, EF0818, bopD, fss1, ebpA, ebpB, ebpC, srtC, ace, fss2, cpsA, cpsB, efaA, EF3023.*
Ef-20	jun-24	Traumatology	ST32	*efaA, srtC, ebpC, ebpB, ebpA, prgB/asc10, fsrA, fsrB, fsrC, gelE, sprE, cpsK, cpsJ, cpsI, cpsH, cpsG, cpsF, cpsE, cpsD, cpsC, cpsB, cpsA, fss2, bopD, fss1, EF3023.*
Ef-21	jun-24	Cardiology	ST585	*fss1, ebpA, ebpB, ebpC, srtC, cpsK, cpsJ, cpsI, cpsH, cpsG, cpsF, cpsE, cpsD, cpsC, cpsB, cpsA, fss2, cylR2, cylR1, cylL, cylS, cylM, cylB, cylA, cylI, sprE, gelE, fsrC, fsrB, fsrA, bopD, EF0485, efaA.*
Ef-22	jun-24	Plastic Surgery	ST32	*bopD, srtC, ebpC, ebpB, ebpA, prgB/asc10, fsrA, fsrB, fsrC, gelE, sprE, fss1, cpsK, cpsJ, cpsI, cpsH, cpsG, cpsF, cpsE, cpsD, cpsC, cpsB, cpsA, fss2, efaA*
Ef-23	jun-24	Thoracic and Cardiovascular Surgery	ST476	*EF3023, ebpA, ebpB, ebpC, srtC, efaA, fss2, cpsA, cpsB, cpsC, cpsD, cpsE, cpsF, cpsG, cpsH, cpsI, cpsJ, cpsK, fss1, bopD.*
Ef-24	jun-24	Neurosurgery	ND	*fss1, cpsK, cpsJ, cpsI, cpsH, cpsG, cpsF, cpsE, cpsD, cpsC, asa1, cpsA, cpsB, cylI, cylA, cylB, cylM, cylS, cylL, cylR1, cylR2, sprE, gelE, fsrC, fsrB, fsrA, EF0818, bopD, prgB/asc10, EF3023, fss2, srtC, ebpC, ebpB, ebpA, efaA*

Figure S1. Antibiotic resistance profile of Enterococcus faecalis non-susceptible to linezolid isolates.

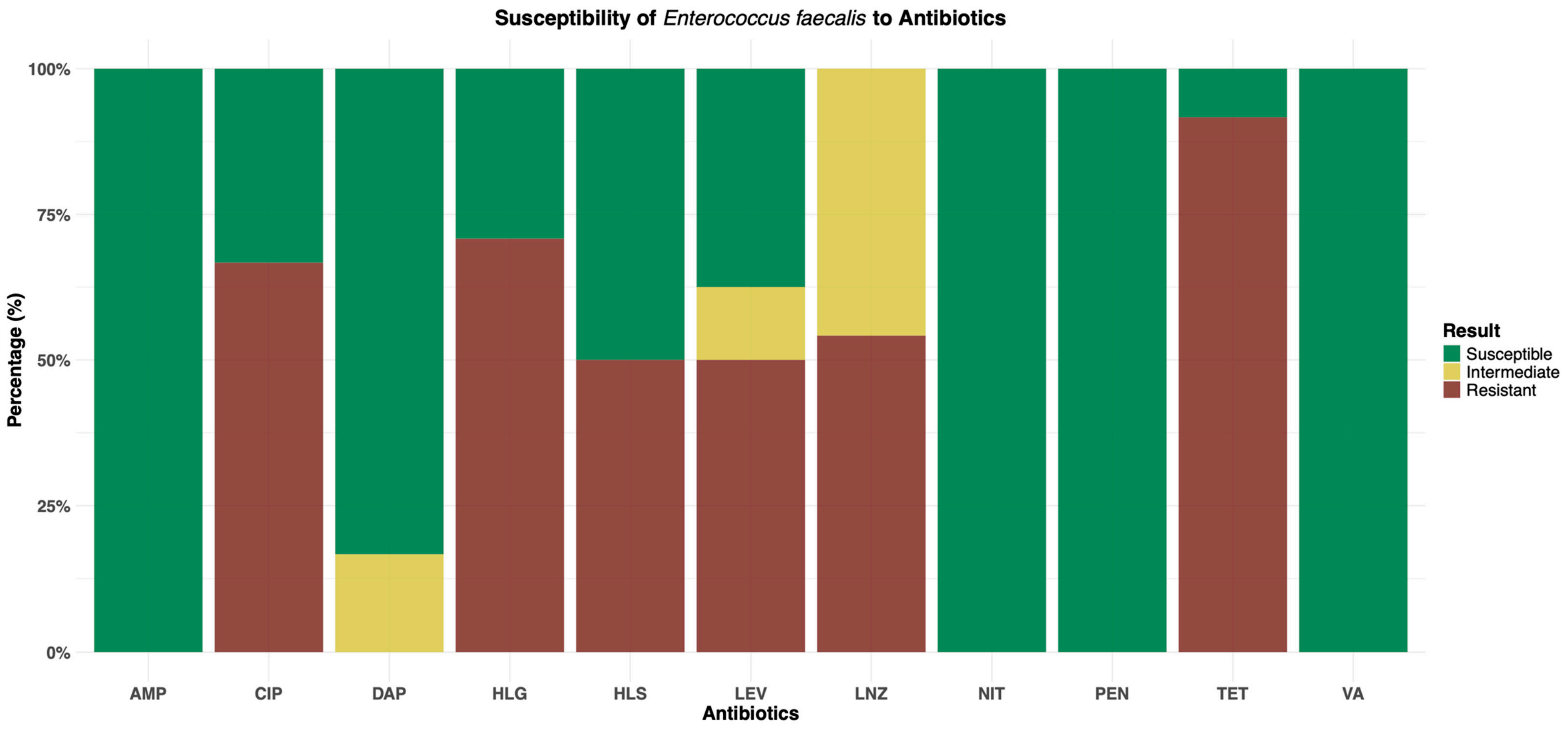

Supplement: Supplementary file 1 [file microorganisms-13-00684-s001.zip › microorganisms-3533814-supplementary.pdf]
